# Ground–state structure of semiconducting and superconducting phases in xenon carbides at high pressure

**DOI:** 10.1038/s41598-019-39176-4

**Published:** 2019-02-21

**Authors:** Thiti Bovornratanaraks, Prutthipong Tsuppayakorn-aek, Wei Luo, Rajeev Ahuja

**Affiliations:** 1 Extreme Conditions Physics Research Laboratory (ECPRL) and Physics of Energy Materials Research Unit (PEMRU), Department of Physics, Faculty of Science, Chulalongkorn University, 10330 Bangkok, Thailand; 2 Thailand Center of Excellence in Physics, Commission on Higher Education, 328 Si Ayutthaya Road, Bangkok, 10400 Thailand; 3 Condensed Matter Theory Group, Department of Physics and Astronomy, Uppsala University, Box 516, S-751 20 Uppsala, Sweden; 4 Department of Materials and Engineering, Applied Materials Physics, Royal Institute of Technology (KTH), SE-100 44 Stockholm, Sweden

## Abstract

The ‘missing Xe paradox’ is one of the phenomena at the Earth’s atmosphere. Studying the ‘missing Xe paradox’ will provide insights into a chemical reaction of Xe with C. We search the ground–state structure candidates of xenon carbides using the Universal Structure Predictor: Evolutionary Xtallography (USPEX) code, which has been successfully applied to a variety of systems. We predict that XeC_2_ is the most stable among the convex hull. We find that the *I*$\bar{4}$2*m* structure of XeC_2_ is the semiconducting phase. Accurate electronic structures of tetragonal XeC_2_ have been calculated using a hybrid density functionals HSE06, which gives larger more accurate band gap than a GGA–PBE exchange-correlation functional. Specifically, we find that the *I*$\bar{4}$2*m* structure of XeC_2_ is a dynamically stable structure at high pressure. We also predict that the *P*6/*mmm* structure of XeC_2_ is the superconducting phase with a critical temperature of 38 K at 200 GPa. The ground-state structure of xenon carbides is of critical importance for understanding in the missing Xe. We discuss the inference of the stable structures of XeC_2_. The accumulation of electrons between Xe and C led to the stability by investigating electron localization function (ELF).

## Introduction

The ‘missing Xe paradox’ is one of the most charming in the Earth’s atmosphere, scientists have been ongoing to great effort to finding the cause of the missing Xe. It is known that the possibility of Xe escaping from the Earth’s atmosphere^1^, the majority of scientist accept that Xe may be hidden in the interior of the Earth^2,3^. The several models for a Xe basin have been proposed since the missing Xe could be contained in the Earth’s inner core has not yet been responded. Attempts to capture Xe in reactivity with elemental metals is observed. Zhu *el al*.^4^ have elucidated that Xe can capture in the Earth’s inner core and have proposed that it would have to form chemically stable compounds with Fe/Ni. They have predicted Xe with Fe/Ni using the Crystal Structure Analysis by Particle Swarm Optimization (CALYPSO) and the *ab initio* random structure searching (AIRSS). They also show that the Xe can capture with with Fe/Ni. In addition, they suggested that the revealed reactivity of Xe with Fe/Ni under the conditions in the Earth’s core not only assists in deciding the missing Xe paradox. Moreover, the revealed reactivity of Xe with O has been found^5^. Two oxides, Xe_3_ O_2_ and Xe_2_ O_5_ phases have synthesized by laser-heated diamond anvil cells as well as *ab initio* calculations coupled with the AIRSS calculation successfully predicted their structures and stability. This may be a general trend in compounds formed under high compression. The understanding of the Earth’s inner core is accepted to be composed originally 8–12% of other light elements (H, C, O, Si, and S)^6^. This is suggested that there might be a significant amount of C in the Earth’s inner core, and the likely stoichiometries of the most stable Fe–C compounds at high pressure^7^.

In this study, we select Xe–C compound as an example to address these problems. We report a predicted high-pressure stabilization of Xe–C compound by the Universal Structure Predictor: Evolutionary Xtallography (USPEX) code. A variety of advance computational methods have been used to identify the thermodynamics stability, dynamics stability, and electronic structure. In addition, a chemical reaction of Xe with C is given way to the relatively open the existence of Xe through the electron localization function. The methods can observe the missing Xe. This is a unique case that Xe is found to be stable in Xe–C compound.

## Results and Discussion

**Figure 1:**
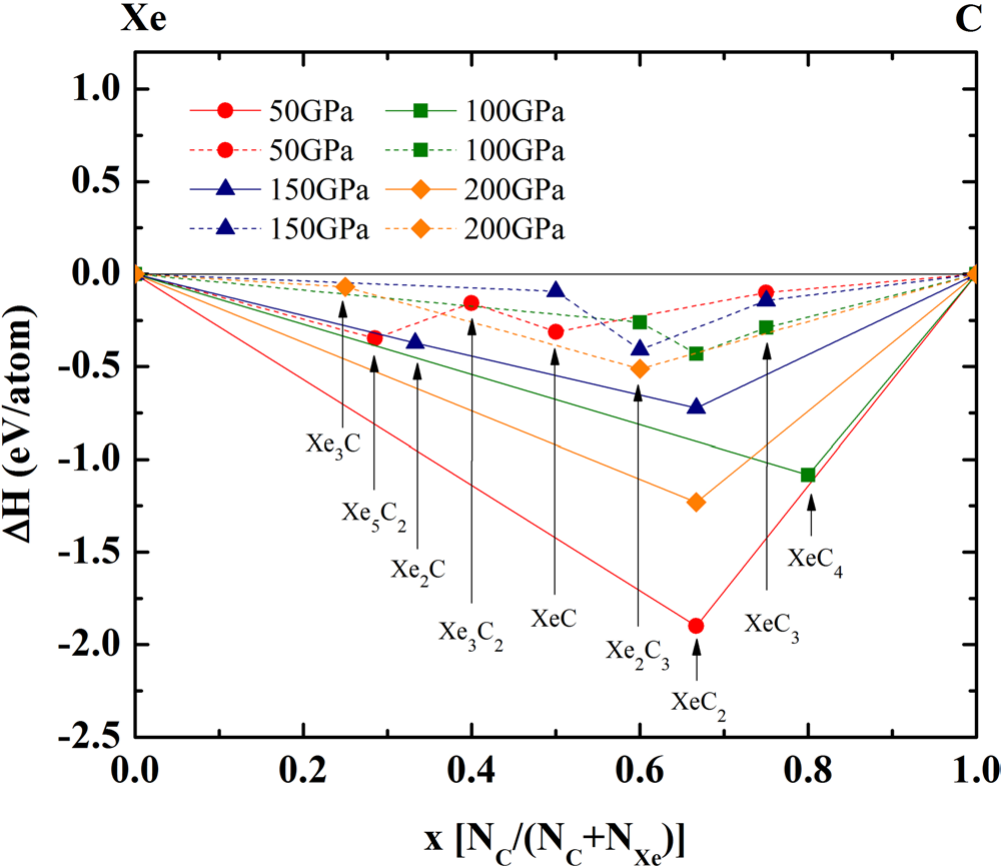
Stability of new xenon carbides. Convex hull diagram for the Xe-C compounds at selected pressures. At a given pressure, the compounds located on the convex hull are thermodynamic stable at *T* = 0 K.

Based on our ground-state searches, we predicted stable structure in Xe–C compound as shown in Fig. 1. The ground-state structure is studied by calculating the enthalpy of formation per atom of Xe and C with respect to their separated counterparts, they are plotted against the fraction x of C atoms on a convex hull diagram. The convex hull diagram of Xe–C compound shows that the stable and meta-stable structure by examining a given pressure connects the phases that are stable against decomposition into other stoichiometries of Xe–C. Structures on the hull indicated by solid line are stable. XeC_2_, Xe_2_ C and, XeC_4_ have found to be stable Xe-C compounds. While structures on the revealed by dash line are meta-stable structure. Xe_3_ C, Xe_5_ C_2_, Xe_3_ C_2_, XeC, Xe_2_ C_3_, and XeC_3_ have found to be the meta–stable Xe–C compounds. We find that XeC_2_ with space group *I*$\bar{4}$2*m* is the most stable structure at 50 GPa.

At pressure 50 GPa, the optimized structural parameters for the tetragonal structure are a = 3.471 Å and c = 18.196 Å with Xe atoms located at 2*b* symmetry site (0.5, 0.5, 0), Xe atoms located at 4e symmetry site (0, 0, 0.887) and C atoms located at 8i symmetry site (0.865, 0.134, 0.715), and C atoms located at 4 *d* symmetry site (0, 0.5, 0.75) and at pressure 200 GPa, the optimized structural parameters for the hexagonal structure are a = 2.539 Å and c = 4.421 Å with Xe atoms located at 1 *a* symmetry site (0, 0, 0) and C atoms located at 2*d* symmetry site (0.333, 0.667, 0.5) as can be obtained from a convex hull of comparative stability.

**Figure 2:**
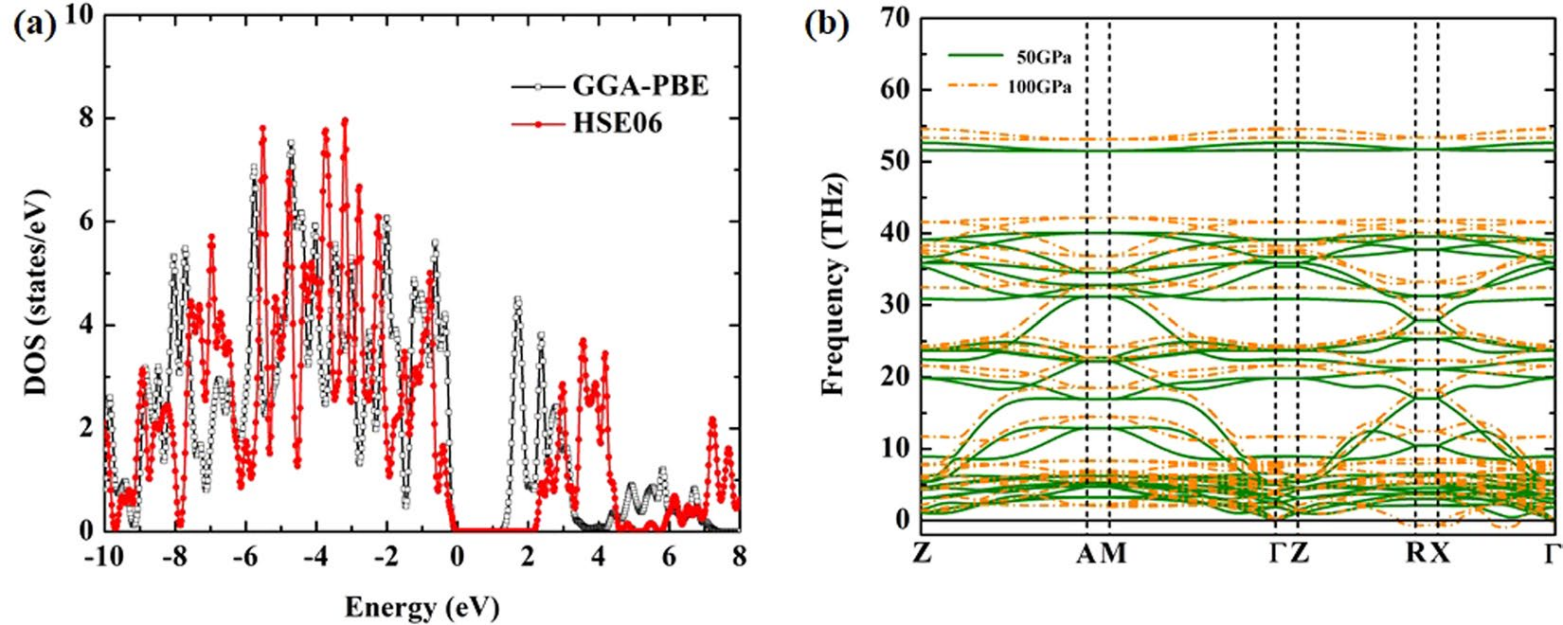
(**a**) Total density of states for two different exchange-correlation functionals: GGA-PBE and HSE06 at 50 GPa and (**b**) harmonic (*T* = 0 K) phonon band structures of tetragonal XeC_2_ structure at 50 and 100 GPa.

The electronic structure of tetragonal XeC_2_ is studied, we found that it is a semiconductor with a band gap of 1.32 eV using the GGA–PBE semi–local density functional at 50 GPa (Fig. 2a). With increasing pressure, the band gap of the XeC_2_ structure is decreased. Above 100 GPa the band gap of tetragonal XeC_2_ has closed. This is because the GGA–PBE underestimates the band gap at high pressure, in order to enhance the band gap of tetragonal XeC_2_, the HSE06 screened Coulomb functional is used. We described in detail the electronic band structure of tetragonal XeC_2_ structure derived from the HSE06 method, however, the XeC_2_ structure with space group *I*$\bar{4}$2*m* has a large unit cell; therefore, the HSE06 calculation of the XeC_2_ structure is more computationally demanding. Hence, the geometry of the XeC_2_ structure has been optimized with the GGA–PBE and then we employed the HSE06 method for the electronic band structure. We found that the band gap of the XeC_2_ is 2.20 eV, which generally gives larger more accurate band gap than the GGA–PBE exchange–correlation functional. We have also presented the band gap of the tetragonal XeC_2_ as can be seen in Table 1.

**Table 1:** Calculated the band gap for XeC_2_ using the GGA–PBE and the HSE06.

Methods	Band gap (eV)		
	P = 50 GPa	P = 75 GPa	P = 100 GPa
GGA–PBE	1.32	1.15	0.71
HSE06	2.20	1.81	1.39

**Figure 3:**
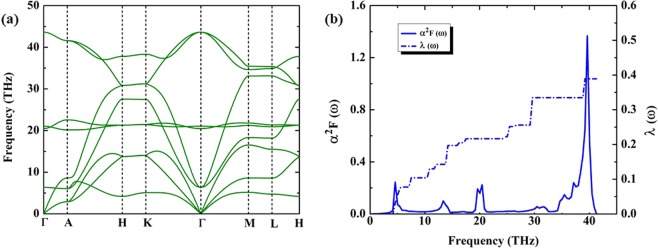
(**a**) Harmonic (*T* = 0 K) phonon band structures of hexagonal XeC_2_ structure at 200 GPa and (**b**) spectral function *α*^2^F (yellow shaded area) and the integral of the spectral function up to frequency *ω* (dash dot line).

To investigate the dynamically stable of XeC_2_ from the harmonic phonon band structures, the calculated phonon band structure of tetragonal XeC_2_ structure revealed that it is stable at 50 GPa. With increasing pressure, the imaginary frequencies in the Brillouin zone of the tetragonal XeC_2_ structure are emerged at 100 GPa (Fig. 2b). We strongly suggested that the tetragonal XeC_2_ structure transforms into a new phase due to it is found to be dynamically unstable. One of the notable features predicted to form on a convex hull diagram, we found that XeC_2_ with space group *P*6/*mmm* is the ground-state structure at 200 GPa. The general expectation is met, we examined the dynamical stability of the *P*6/*mmm* structure which is found to be stable at pressure 200 GPa because of the lacking of any imaginary frequencies as shown in Fig. 3a. This remarkable result of the *P*6/*mmm* structure, the spectral function *α*^2^F for the *P*6/*mmm* structure shown that the electron-phonon coupling (EPC) distributes over the entire this frequency range as can be seen Fig. 3b. The situation is shown that the integral of the spectral function *λ* of the *P*6/*mmm* structure is 0.38 and the calculated logarithmic average of phonon frequency *ω*_*log*_ is 622 K. By using the Coulomb pseudopotential μ^*^ = 0.10 for estimation superconducting transition temperature *T*_*c*_, the *P*6/*mmm* structure predicts to give the maximum *T*_*c*_ of around 38 K at 200 GPa.

**Figure 4:**
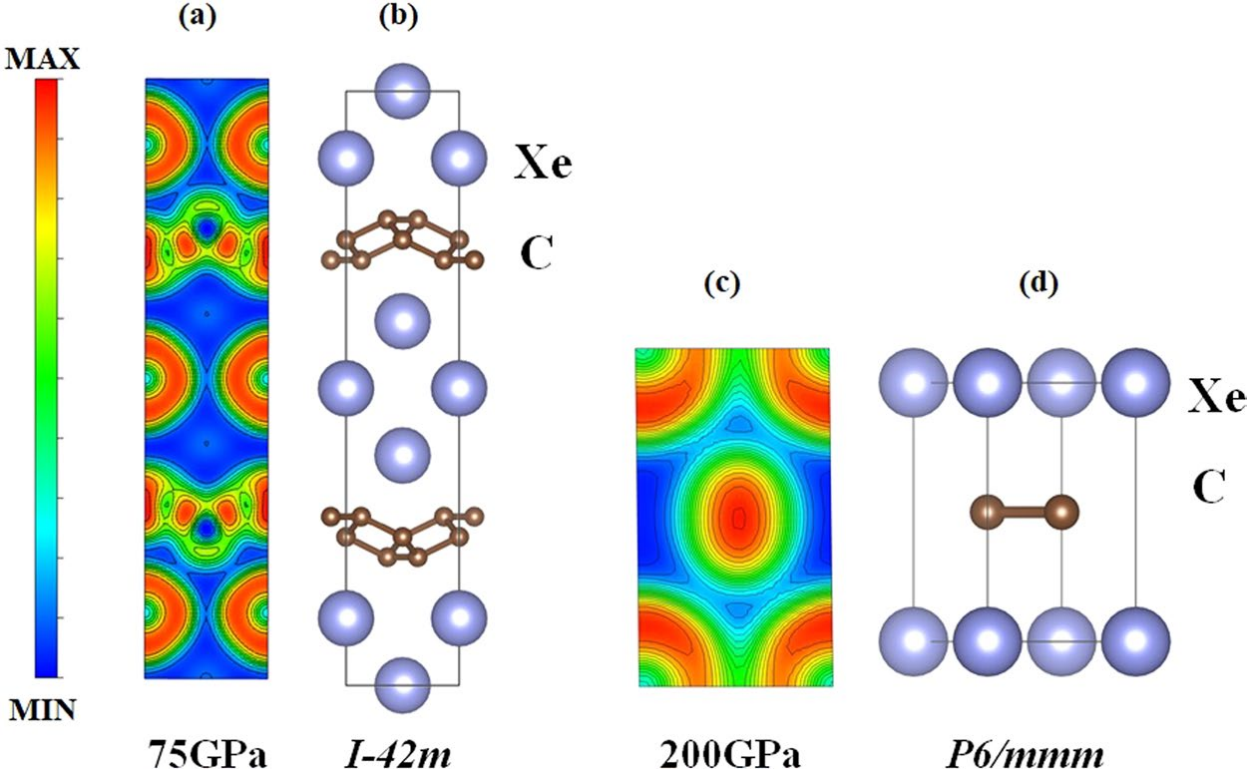
(**a**) Projections of the charge density in the (100) plane and structure of tetragonal XeC_2_ at 75 GPa. (**b**) Structure of XeC_2_ illustrating the tetragonal structure with space group *I*$\bar{4}$2*m*. (**c**) Projections of the charge density in the (100) plane and structure of hexagonal XeC_2_ at 200 GPa. (**d**) Structure of XeC_2_ illustrating the hexagonal structure with space group *P*6/*mmm*.

The victorious prediction of Xe–C compounds in this study, the enthalpy has shown that Xe (the ‘missing Xe paradox’) can be formed into tetragonal XeC_2_. Xe may be hidden in the interior of the Earth as the Earth’s inner core is accepted to be composed originally 8–12% of other light elements. Interestingly, C is one of other light elements and C can exist in a diverse number of forms due to its capability to form the *sp*^*n*^ (n = 1; 2; 3) hybridized bonds. C changes its hybridization state with increasing pressure. The formation of *sp*^*n*^ hybridized bonds can be explained by transformation of carbon’s *sp*^2^ hybridization in the graphite structure. The *p* electron transfered from the *sp*^2^ (graphite structure) into the *sp*^3^ (the diamond structure) hybridizations under high pressure. Moreover, superhard graphite is shown that a half of C atoms converted the *σ* bond to *π* bonds^8^. Hence the tendency of C to change hybridization evident also in carbon compounds. In Fig. 4a, we proposed the formation of *sp*^3^ hybridized bonds by analyzing the electron localization function (ELF) in the (100) plane. On pressure increase, 75 GPa, the ELF in the (100) plane shown the formation of *sp*^3^ hybridized bonds of C the formation of a bonding between C and Xe atoms which there is a weak bonding between Xe atoms forming the C layer. Moreover, we have obtained the *P*6/*mmm* structure from the ground–state search at 200 GPa. We now present the *P*6/*mmm* structure by considering the ELF in the (100) plane (Fig. 4b). It indicated that the distribution of electrons is localized around Xe and C atoms and shown the formation of *sp*^3^ hybridized bonds of C the formation of a bonding between C and Xe atoms which there is a weak bonding between Xe atoms forming the C atoms. The accumulation of electrons between Xe and C led to the stability of the *P*6/*mmm* structure. According to the ELF study, the effect of pressure displayed that the *p* electron transfered from the *sp*^2^ into the *sp*^3^. In particular, C opens up the half-filled 2*p* state as valence state, thus the charge transfer took place between the C 2*p* states and the Xe 5*p* states. Moreover, Mulliken’s analysis of electron density reveals the amounts of transferred charge in XeC_2_ is 0.68 *e*/Xe. This remarkable result displays an orbital hybridization, which is caused by the application of pressure. The charge-transfer phenomenon supports a possibility of the stabilization of the stable structure of XeC_2_.

## Conclusion

In conclusion, we predict a new high-pressure phase of Xe–C compound by the *ab initio* calculations combined with the USPEX. The tetragonal XeC_2_ structure is predicted to be ground state structure and dynamically stable at 50 GPa. The formation of *sp*^*n*^ hybridized bonds indicates the formation of *sp*^3^ hybridized bonds of C the formation of a bonding between C and Xe atoms. This is because a tendency of carbon to change hybridization is also evident in carbon compounds, which is required for xenon. The ‘missing Xe paradox’ can be formed in carbon compounds under high pressure. Using the HSE06 for band gap calculation gives larger more accurate band gap than the GGA–PBE. We show that the formation of XeC_2_ under pressure is common and examine the possibility of superconductivity in relation to fundamental phases of materials science. These observations are of fundamental importance of the semiconducting–superconducting phase transition.

## Methods

We presented a novel phase using a structural prediction USPEX code^9,10^. To get the most stable structure of Xe–C compound, we searched for its lower enthalpy’s phase based on USPEX code in combined with Vienna *ab initio* simulation package (VASP) package. The GGA–PBE^11^ for the exchange-correlation functional to density functional theory. We employed the projector augmented wave (PAW) method^12^. The PAW potential with an 8-electron (5 s^2^ 5p^6^) for Xe and 4-electron (2 s^2^ 2p^2^) for C have been employed. The pseudocore radius of Xe is 2.5 bohr and of C is 1.5 bohr, which are small enough that the overlap of spheres will not occur. We performed the prediction simulations using USPEX code for Xe-C compound (XeC, XeC_2_, XeC_3_, XeC_4_, Xe_2_ C_3_, Xe_3_ C_2_, Xe_2_ C, Xe_3_ C, and Xe_5_ C_2_) containing one, two, three, and four formula units in the simulation cell at 50, 100, 150, and 200 GPa, respectively. To get the most stable structure of Xe–C compound, a plane waves basis set up to a cutoff energy of 800 eV and the initial BZ sampling grid of spacing 2 *π* × 0.01 Å^−1^. The parameters of all structures are fully relaxed using a conjugate gradient scheme. All structures are relaxed at each pressure until the Hellman-Feynman forces became less than 10^−3^ eV/Å. We predicted the stable structure of Xe–C compound in the form of a convex hull of comparative stability by examining the enthalpy *H* of each formula unit under the relevant pressure. All enthalpies *H* are given at the same pressure and zero temperature. At a given pressure, the Xe–C compound located on the convex hull are thermodynamically stable against decomposition to any other binaries or the elements, while the compounds above the convex hull are the meta-stable structure.

The phonon calculations are carried out by using a supercell approach within the PHONOPY code^13^ combined with VASP code. To look at the semiconducting phase of Xe–C compound, one has to go beyond a standard DFT in order to get well defined excited state properties. We employed the hybrid functional of Heyd, Scuseria, and Ernzerhof (HSE06)^14,15^ to verify the results of band structures with the GGA-PBE for the exchange-correlation functional. A plane wave basis set 800 eV and the initial BZ sampling grid of spacing 2 *π* × 0.04 Å^−1^ are used.

We calculated the EPC with density functional perturbation theory^16^. The plan waves basis set is expanded with a kinetic energy cutoff of 60 Ry. The calculation studies presented here are based on the GGA–PBE. We employed GGA–PBE method as implemented in Quantum Espresso (QE)^17^. The BZ integrations in the electronic and phonon calculations are performed using MP meshes. Both the meshes of k-points for electronic states and the meshes of phonons are used in these calculation. For the *P*6/*mmm* structure, individual phonon calculations are performed on the first BZ on 4 × 4 × 2 q-meshes with a 12 × 12 × 8 k-points mesh. The EPC matrix elements are computed in the first BZ on 4 × 4 × 2 q-meshes using individual EPC matrices obtained with a 24 × 24 × 16 k-points mesh.

1 $${T}_{c}=\frac{{\omega }_{log}}{1.2}\exp \,[-\frac{\mathrm{1.04(1}+\lambda )}{\lambda -{\mu }^{\ast }\mathrm{(1}+0.62\lambda )}],$$

where *ω*_*log*_ is the averaged phonon frequency. We used effective Coulomb interaction parameter μ^*^ = 0.10. It is assumed by the original Allen-Dynes formula^18^.

LaTeX formats citations and references automatically using the bibliography records in your.bib file, which you can edit via the project menu. Use the cite command for an inline citation, e.g.^1–19^.
